# Antidepressant prescribing patterns in Australia

**DOI:** 10.1192/bjo.2022.522

**Published:** 2022-06-30

**Authors:** Gin S. Malhi, Mustafa Acar, Mahsa H. Kouhkamari, Tzu Hsiang Chien, Prabhjot Juneja, Sinthuja Siva, Bernhard T. Baune

**Affiliations:** Academic Department of Psychiatry, Kolling Institute, Northern Clinical School, Faculty of Medicine and Health, The University of Sydney, St Leonards, New South Wales, Australia; CADE Clinic, Department of Psychiatry, Royal North Shore Hospital, Northern Sydney Local Health District, St Leonards, New South Wales, Australia; and Department of Psychiatry, University of Oxford, Oxford, UK; Janssen-Cilag Pty Ltd, North Ryde, New South Wales, Australia; Prospection, Redfern, New South Wales, Australia; Janssen-Cilag Pty Ltd, North Ryde, New South Wales, Australia; Prospection, Redfern, New South Wales, Australia; Janssen-Cilag Pty Ltd, North Ryde, New South Wales, Australia; Department of Psychiatry, University of Münster, Albert-Schweitzer-Campus 1, Münster 48149, Germany; Department of Psychiatry, Melbourne Medical School, The University of Melbourne, Melbourne, Australia; and The Florey Institute of Neuroscience and Mental Health, The University of Melbourne, Parkville, Victoria, Australia

**Keywords:** Antidepressants, depressive disorders, clinical practice guidelines, prescribing patterns, real-world evidence

## Abstract

**Background:**

The Royal Australian and New Zealand College of Psychiatrists clinical practice guidelines for mood disorders (MDcpg^2015^ and MDcpg^2020^) provide evidence-based and consensus-based recommendations for managing mood disorders.

**Aims:**

We examined Australian real-world prescribing habits to determine whether management in clinical practice aligned with MDcpg^2015^ recommendations.

**Method:**

A retrospective analysis of a cohort of patients ≥16 years old who had been dispensed a Pharmaceutical Benefits Scheme (PBS)-listed antidepressant between July 2013 and June 2019 was conducted using Australian Commonwealth Department of Human Services PBS 10% sample data.

**Results:**

Between July 2013 and June 2019, 239 944 patients in Australia commenced antidepressant treatment. Of these, 22% (52 694 patients) received a second treatment (a new class of treatment after a period of discontinuation or additional antipsychotic therapy) and 6% (15 741 patients) received a third treatment. Patients were initially prescribed primarily selective serotonin reuptake inhibitors (SSRIs; 52% of prescriptions) or tricyclic antidepressants (TCAs; 25%), even though TCAs are not recommended for first-line treatment. Fewer than one-quarter of patients were prescribed serotonin–noradrenaline reuptake inhibitors (13%) or other agents (10%). General practitioners (GPs) were more likely to initiate TCAs than psychiatrists (22% *v*. 7%).

Once initiated, the overall median time patients remained on treatment was 4.5 months; this was highest with SSRIs (5.8 months) and lowest with TCAs (0.9 months).

**Conclusions:**

First-line prescribing broadly follows guidelines. GP and psychiatrist prescribing patterns differ, perhaps reflecting different patient groups and the need to tailor treatment to individuals. Future guidelines should aim to capture the different presentations and complexity of depression.

The 12 month prevalence of major depressive disorder (MDD) varies across countries, but each year in Australia approximately a million people suffer from clinical depression.^1^ The recent comprehensive Royal Australian and New Zealand College of Psychiatrists clinical practice guidelines for the management of mood disorders (MDcpg^2021^) build on an earlier set of guidelines published in 2015 (MDcpg^2015^) and outline the optimal selection and subsequent sequencing and augmentation of pharmacotherapies for the treatment of major depression.^2,3^ These guidelines, based on scientific evidence and expert clinical consensus, are intended for clinical use by psychiatrists, psychologists, primary care physicians and others with an interest in mental healthcare. A summary of the guidelines has been provided that is likely to be particularly useful for community management and those in training.^4^

## Guideline recommendations

The overarching aim of treatment is to achieve remission of depressive symptoms with eventual functional recovery and ideally the future development of resilience. Psychological therapy is recommended initially, especially for mild to moderate depression, and prioritised as part of *Actions* (treatments that need to be undertaken wherever possible). This can be achieved alongside pharmacotherapy even in cases of more severe or chronic presentations – although engagement may be limited in instances of severe melancholia, for example.^2,4^ First- and second-line pharmacotherapy options are specified in the MDcpg^2015^ guidelines, as well as adjunctive and augmentation therapies (Table 1). Although the more recent guidelines do not specify this sequencing, the emphasis on trialling a number of medications and using strategies such as increasing the dose of antidepressant medication and augmentation of a partial response are retained and captured in the MiDAS (medication, increase dose, augment, switch) paradigm.^2^

**Table 1:** Antidepressants recommended in Australia by line and class^3^

Class	Agents
First line	
Selective serotonin reuptake inhibitor	Citalopram, escitalopram, fluoxetine, fluvoxamine, paroxetine, sertraline
Noradrenaline reuptake inhibitor	Reboxetine
Noradrenaline and specific serotonergic antidepressant	Mianserin, mirtazapine
Melatonergic agent	Agomelatine^a^
Noradrenaline–dopamine reuptake inhibitor	Bupropion^a^
Second line	
Serotonin–noradrenaline reuptake inhibitor	Desvenlafaxine, venlafaxine, duloxetine, milnacipran^a^
Tricyclic antidepressant (non-selective monoamine reuptake inhibitors)	Amitriptyline, clomipramine, dothiepin, imipramine, nortriptyline, trimipramine^a^, doxepin
Serotonin modulator	Vortioxetine^a^
Monoamine oxidase inhibitor (MAOI)	Phenelzine, tranylcypromine
Reversible MAOI	Moclobemide
Adjunctive	
Serotonin antagonist and reuptake inhibitor	Trazodone^a^
Augmentation	
	Lithium^a^
Second-generation antipsychotics	Aripiprazole^a^, olanzapine^a^, quetiapine,^a^ risperidone^a^

a. Not available on the Pharmaceutical Benefits Scheme for the treatment of depression.

Initial antidepressant pharmacotherapy should be trialled for at least 3 weeks at the optimal dose. If the response is inadequate, the following may be considered: a dose increase, augmentation of therapy where some response has occurred or a switch to a different class of antidepressant.

Various factors influence both the initial and subsequent choices of pharmacotherapy, including the possibility and nature of side-effects and drug–drug interactions, especially for adolescents, the elderly and pregnant or breastfeeding women.^3^ The toxicity of the chosen therapy in overdose should also be considered, particularly for those at risk of suicide.

Treatment switches may be made for lack of response, poor response or non-adherence secondary to tolerability issues.^4^ In general, however, newer generations of antidepressants are better tolerated than tricyclic antidepressants (TCAs) or monoamine oxidase inhibitors (MAOIs), and switching usually occurs because of lack of response.

## Study objective

The objective of this study was to examine the antidepressant prescribing patterns in Australian real-world clinical practice. In particular, we aimed to map the prescribing habits of clinicians and determine how closely, if at all, they matched the recommendations made in treatment guidelines. For this we used the MDcpg^2015^ as these were in place at the time of our analysis.^3^ We decided to focus on the antidepressant treatment being prescribed, as well as the maintenance of pharmacotherapy (persistence with medication) and the use of various strategies such as the rate and timing of dose escalation, switching, combination and augmentation. We note, however, that there are many alternative guidelines that can be used by clinicians, such as the CANMAT guidelines.^5^

## Method

This retrospective cohort analysis was conducted using the Australian Commonwealth Department of Human Services Pharmaceutical Benefits Scheme (PBS) 10% sample data. The PBS data-set is a systematic random 10% sample of dispensing of prescription medicines subsidised by the Australian government and is considered to be representative of the Australian population.^6^ It includes PBS-subsidised prescriptions from community pharmacies, private hospitals and discharging patients and out-patients at public hospitals. The 10% sample is made available by the Australian government to approved data custodians for the purposes of research. In this study, Prospection acted as the data custodian.

Longitudinal data from July 2012 to June 2019 were analysed in this study. Data prior to 2012 were not analysed because general patient co-payment prescription data were not available before this time. This study and publication of subsequent results were approved by Services Australia (External Request Evaluation Committee Approval Number RMS1927).

### Data selection

Data were extracted for patients aged 16 years or older who had a PBS-listed antidepressant dispensed between July 2013 and June 2019. Patients were excluded from the study if they had a PBS-listed antidepressant or antipsychotic dispensed in the period July 2012 to June 2013. This was an attempt to include patients’ first prescriptions of antidepressants and exclude patients who may have been treated for psychosis or related conditions. Data were extracted for all antidepressants approved by the Australian Therapeutic Goods Administration for treatment of MDD and government reimbursed under the PBS. Most of the medications included in the analysis had restricted benefits under the PBS for MDD, depression or MDD mixed, although some medications were not restricted for depression (amitriptyline, clomipramine, dothiepin, doxepin, imipramine and tranylcypromine; see Supplementary Table 1 available at https://doi.org/10.1192/bjo.2022.522). Drugs used as mood stabilisers, such as lithium, carbamazepine, oxcarbazepine, valproic acid and lamotrigine, do not have any restrictions under the PBS and were not included as antidepressants in this study.

Extracted data included year of birth, sex, state where the prescription was filled, PBS item code and drug dispensing date (date of supply). Variables from these used for analysis included age at initiation (dispensing year minus year of birth) for persistence data and age at dispensing otherwise, molecule and indication (both inferred from the PBS item code and its corresponding authority information) and line of therapy (calculated based on order of therapy). Variables not included in the data source included reason for initiation, reason for discontinuation, reason for switch and diagnosis (although this could be inferred from ‘indication’).

The first prescription for an antidepressant was considered the ‘index prescription’. First antidepressant treatment was defined as a prescription for antidepressant therapy, where no antidepressant prescribing had occurred in the previous 12 months. Patients were not included if an antipsychotic was dispensed prior to or at the same time as the first identified antidepressant dispensation, in order to omit therapy that resembled the management of psychosis or psychosis-related conditions.

### Statistical analysis

Antidepressants were analysed by class of medication (Table 1) rather than as individual medications to align with MDcpg^2015^ and to allow better detection of trends in prescribing.

- The first antidepressant treatment was called ‘treatment 1’.
- A patient was considered to have discontinued antidepressant therapy if there was a period of 6 months during which no further antidepressant was dispensed.
- A patient was considered to have commenced their second antidepressant treatment ‘treatment 2’ if they commenced any new class of antidepressant treatment (including their initial therapy) after meeting the discontinuation criterion, or if a patient switched to a new class of treatment, or if an antipsychotic or new class of antidepressant was added (augmentation or combination therapy).
- Increase in dose was estimated to have occurred when there was an increase in the dose strength of medication supplied e.g. from 10 mg tablets to 40 mg tablets. Only a patient's first increase in dose was included in the analysis. This method was used as a proxy for dose increase, as prescribed dose was not available in the PBS data.
- A patient was considered to be on combination therapy if they had two or more antidepressants from two or more concurrent prescriptions. A patient was considered to be on augmentation therapy if an antidepressant and an antipsychotic had been dispensed in two or more concurrent prescriptions.

When treatment switch, combination or augmentation occurred, the date at which the patient was considered to have changed treatment was the date that the new prescription was dispensed.

Treatment persistence was defined as the time (in consecutive months) from commencement of treatment until the date of treatment change. Treatment persistence was estimated using Kaplan–Meier methods, stratified by the treatment number and by drug class. Pairwise comparisons of the Kaplan–Meier estimates were conducted using log-rank tests or relative risk (RR) comparing persistence at 12 months, with *P* < 0.05 considered statistically significant. All analyses were conducted using Prospection's proprietary PharmDash software.

## Results

Between July 2013 and June 2019, 239 944 patients in the PBS 10% sample commenced treatment with an antidepressant. Of these, 22% (52 694 patients) went on to receive a second treatment, 5% (12 877 patients) received a third treatment and only 1% (2864 patients) received a fourth or later treatment.

Fifty-six per cent of patients in the sample were female (Table 2). The proportions of patients by age category and by state were approximately proportional to the distributions in the Australian population.^7^

**Table 2:** Demographic characteristics of people in the 10% Pharmaceutical Benefits Scheme sample who commenced treatment with an antidepressant between July 2013 and June 2019

Characteristic		Proportion of sample (%)
Sex	Female	55.9
	Male	44.1
Age group^a^	Over 65 years	23.7
	56 to 65 years	18.1
	46 to 55 years	17.9
	36 to 45 years	17.7
	26 to 35 years	15.5
	16 to 25 years	15.4
State	New South Wales	29.6
	Victoria	23.6
	Queensland	22.3
	South Australia	7.4
	Western Australia	11.7
	Tasmania	2.6
	Australian Capital Territory	1.9
	Northern Territory	0.9

a. Age at dispensing; therefore, patients may appear in more than one age group.

### Prescribing patterns

Treatment 1 prescriptions were most commonly selective serotonin reuptake inhibitors (SSRIs; *n* = 1 259 140; 52% of patients), followed by TCAs (*n* = 594 040; 25% of patients) and serotonin–noradrenaline reuptake inhibitors (SNRIs; *n* = 312 020; 13% of patients). Similarly, treatment 2 initiations included prescriptions for SSRIs (*n* = 249,550; 38% of patients), SNRIs (*n* = 175,980; 27% of patients), noradrenergic and specific serotonergic antidepressants (NaSSAs; *n* = 120 090; 18% of patients) and TCAs (*n* = 109 210; 17% of patients). The proportions of patients initiating each class of antidepressant remained stable during the study for treatment 1 and treatment 2. Overall, the most commonly prescribed individual antidepressants were amitriptyline (19%), escitalopram (19%), sertraline (15%), mirtazapine (11%) and fluoxetine (7%); together these constituted 71% of all prescriptions. The most commonly prescribed antidepressants remained the same when only patients who were active in the most recent 12 months of the study period were considered.

### Patterns by prescriber type

General practitioners (GPs) were responsible for more than two-thirds of prescriptions (68.4%) and nearly three quarters of first-treatment initiations (74.6%). The proportion of initial prescriptions written by GPs decreased with line of therapy to 58.3% at treatment 3 and later treatments (treatment 3+). The proportion of initial prescriptions by psychiatrists increased from 2.9% at treatment 1 up to 18.3% at treatment 3+. Interns provided 9.7% of treatment 1 initial prescriptions, increasing to 11.7% of initial treatment 3+ prescriptions.

GPs and psychiatrists showed different patterns of prescribing. Psychiatrists tended not to initiate TCAs and most often initiated people on an SSRI (Table 3). GPs also favoured SSRIs but tended to prescribe TCAs more often than psychiatrists (21.9% *v*. 3.6% of treatment 1 prescriptions).

**Table 3:** Proportion of prescriptions by class of antidepressant for each prescriber type at treatment 1, treatment 2, and treatment 3 and later lines of treatment

	Prescriber type (overall proportion of prescribing)	SSRI (%)	TCA (%)	SNRI (%)	NaSSA (%)	RIMA (%)	NRI (%)	Tetracyclic (%)	MAOI (%)
Treatment 1	GP (74.6%)	54.9	21.9	13.3	9.7	0.1	0.0	0.0	0.0
	Psychiatrist (2.9%)	73.1	3.6	12.0	10.0	0.5	0.4	0.2	0.1
	Intern (9.7%)	51.0	23.7	13.4	11.7	0.1	0.0	0.0	0.0
	Other prescribers (11.7%)	33.5	47.4	9.9	9.0	0.0	0.0	0.0	0.0
	Unknown (1.2%)	44.5	30.5	13.1	11.8	0.0	0.0	0.1	0.0
Treatment 2	GP (69.4%)	38.7	15.9	27.0	17.8	0.4	0.2	0.2	0.0
	Psychiatrist (7.7%)	39.7	6.3	30.7	20.1	1.1	1.3	0.6	0.1
	Intern (10.5%)	38.8	15.2	25.7	19.9	0.3	0.1	0.1	0.0
	Other prescribers (11.1%)	30.0	28.9	23.3	17.2	0.2	0.2	0.1	0.0
	Unknown (1.3%)	36.9	18.9	22.7	21.0	0.1	0.4	0.0	0.0
Treatment 3+	GP (58.3%)	29.7	17.0	27.7	24.1	0.9	0.4	0.2	0.0
	Psychiatrist (18.3%)	26.7	11.8	32.7	23.6	1.7	2.3	0.9	0.4
	Intern (11.7%)	29.9	15.2	28.0	25.7	0.6	0.4	0.1	0.1
	Other prescribers (10.2%)	24.5	25.1	26.3	22.8	0.5	0.5	0.3	0.0
	Unknown (1.5%)	29.8	13.7	27.9	26.5	0.3	1.4	0.3	0.3

GP, general practitioner; MAOI, monoamine oxidase inhibitor; NaSSA, noradrenergic and specific serotonergic antidepressant; NRI, noradrenergic reuptake inhibitor; RIMA, reversible monoamine oxidase inhibitor; SNRI, serotonin–noradrenaline reuptake inhibitor; SSRI, selective serotonin reuptake inhibitor; TCA, tricyclic antidepressant.

### Age-related prescribing patterns

SSRIs were the most commonly prescribed antidepressant in all age groups except people aged over 65 years, who were more frequently prescribed TCAs. Of note, the percentage of people receiving SSRIs declined with age from 62.0% in people aged up to 25 years to 33.9% in those aged above 65 years. Prescribing of TCAs and NaSSAs increased with age. The proportion of TCAs increased from 10.6% of prescriptions in people aged up to 25 years to 35.4% of those aged above 65 years, and the proportion of NaSSAs increased from 10.0% to 18.9% from the youngest to oldest age groups.

### Treatment persistence

**Fig. 1:**
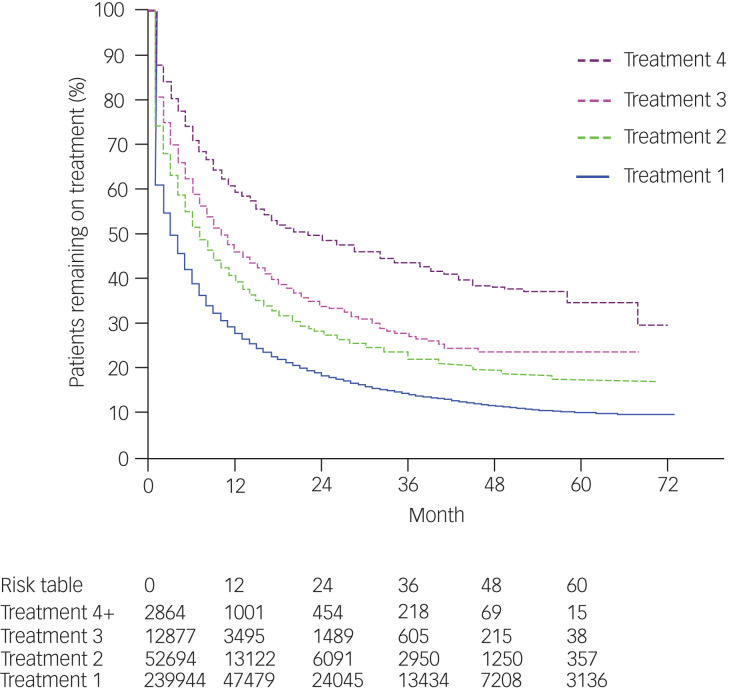
Kaplan–Meier estimates of overall persistence with antidepressant therapy and persistence by treatment number.

Overall median persistence on treatment was 4.5 months, with a third (34%) of people remaining on treatment at 12 months and almost a quarter (24%) continuing to 24 months for all antidepressants (Fig. 1). Overall treatment persistence increased with later therapies (Fig. 1); median persistence increased from 3.0 months for treatment 1, with 28.1% of people remaining on treatment at 12 months, to 22.2 months for treatment 4, with 59.8% of people remaining on treatment at 12 months (RR 0.43, 95% CI 0.41 to 0.44, *P* < 0.001; reference is treatment 1).

Median persistence by class of antidepressant was highest with SSRIs (5.8 months) and lowest with TCAs (0.9 months) (RR for persistence by class of antidepressant with reference to SSRIs: SNRI 1.06, 95% CI 1.04 to 1.07, *P* < 0.001; NaSSA 1.26, 95% CI 1.24 to 1.28, *P* < 0.001; MAOIs 1.33, 95% CI 0.91 to 1.93, *P* = 0.09; reversible monoamine oxidase inhibitor 1.34, 95% CI 1.22 to 1.48, *P* < 0.001; tetracyclic antidepressants 1.45, 95% CI 1.22 to 1.72, *P* < 0.001; TCAs 1.61, 95% CI 1.59 to 1.63, *P* < 0.001; noradrenergic reuptake inhibitors 1.62, 95% CI 1.42 to 1.86, *P* < 0.001). Notably, there were no substantial differences in persistence between treatments within a class.

### Dose changes and augmentation

We estimated that by 12 months 19% of patients had had a dose increase and that by 24 months this had increased to 23%. For patients who had a dose change, the median time to dose increase was 4 months.

For patients within the study sample, switching between classes of antidepressant therapy tended to occur more often than addition of another class, with 6% switching within 6 months of starting treatment compared with 1% initiating combination therapy. Patients receiving SSRIs switched treatment faster and more often within the same class.

For patients who received combination antidepressant therapy, the overall median time to receiving combination therapy was 9 months. Combination therapy was most commonly received by patients who initiated treatment with an NaSSA, SSRI or SNRI. Combination therapy was least likely to be received by patients initiating treatment with TCAs. A NaSSA was most likely to be combined with an SSRI or SNRI, with 1.4% and 0.8% of patients in the study receiving these combinations at 24 months, respectively.

Augmentation with an antipsychotic occurred for 3% of patients at 12 months and 4% at 24 months. At 12 months, patients who received MAOIs were most likely to be receiving augmented therapy (7% of patients), followed by NaSSAs (6%) and tetracyclic antidepressants (5%).

### Duration of treatment-free episode

**Fig. 2:**
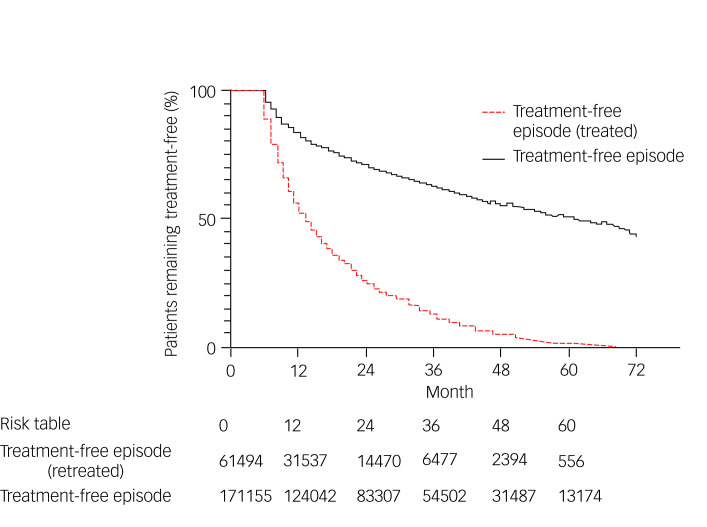
Kaplan–Meier estimates of treatment-free episodes.

Kaplan–Meier estimates of ‘treatment-free’ episodes, where patients had no pharmacological interventions for depression for a period of at least 6 months, were used to determine whether patients subsequently restarted antidepressant therapy and to estimate how long this would take. Over a 70 month period, 36% of patients who discontinued their medication returned to treatment (Fig. 2). Of those who returned to treatment, the median time to return to treatment was 12.8 months.

After discontinuing antidepressant therapy, the median time for patients to restart treatment was longest for those who had received tetracyclic antidepressants (18 months) and shortest for those who had received MAOIs (11 months; no statistically significant difference between median time to restart tetracyclic antidepressants and MAOIs).

## Discussion

The MDcpg^2015^, which were published during the sampling frame of our analysis, recommend first-line treatment with SSRIs, NaSSAs, noradrenaline–dopamine reuptake inhibitors, SNRIs, noradrenergic reuptake inhibitors, melatonin agonists or a serotonin modulator.^3,8^ TCAs and MAOIs were second-line recommendations.^3,8^ We found that SSRIs were the most commonly prescribed treatment overall; however, in contrast to the MDcpg ^2015^ recommendations, TCAs were actually the second most commonly prescribed first-line antidepressant. First-line TCAs were more commonly prescribed by GPs, who prescribed the majority of all first-line antidepressants (77% in our study).

Prescribing of second antidepressant treatments, most commonly SSRIs and SNRIs, also appeared to follow the guidelines.^8^ Psychiatrist prescribing patterns most closely followed the MDcpg^2015^ recommendations.

It is important to note that the data in our analysis relate to patient treatment while the MDcpg^2015^ were in place. These have been supplanted as of 2021, and the new MDcpg^2021^ now recommend that clinicians should select from a list of preferred agents, termed *Choices*, which have been selected as archetypes of classes on the basis of mechanism of action and are differentiated according to tolerability and efficacy.^2^ In addition, the choice of agent must be informed by the clinical profile of the patient. For example, SNRIs and SSRIs are recommended for patients who are particularly experiencing anxiety, and agomelatine and vortioxetine are recommended for patients for whom sleep disturbance is a prominent symptom. It should be noted, however, that anxiety is a common concomitant of low mood that may confound clinical complaints. Further, in clinical practice, anxiety is also often a *forme fruste* of MDD, sometimes preceding its onset by many years.^9^ In addition, although the dosages of ‘antidepressants’ used for the treatment of anxiety disorders are usually more modest than those used in the treatment of clinical depression, there are instances where severe anxiety, especially that marked by obsessive thinking, may necessitate higher doses.

Similar patterns of prescription to those seen in our study were described using claims data in an earlier Australian study.^10^ In that study, from 20 years ago, more than eight in ten antidepressant prescriptions were written by GPs.^10^ There were double the proportion of TCA prescriptions written by GPs compared with psychiatrists (27% *v*. 13% of all antidepressant scripts written), and of all TCA prescriptions, 90% were written by GPs and only 5% by psychiatrists.^10^ In the time since that publication, an Australian Department of Health and Ageing Drug Utilisation Sub-Committee review of psychotropic prescribing between 2000 and 2011 reported decreases in TCA prescribing and increases in SSRI prescribing.^11^ By 2011, 59.2% of antidepressant prescriptions were for SSRIs,^11^ a similar proportion to that observed in our study (where SSRI prescriptions accounted for 57% of GP prescriptions and 56% of psychiatrist prescriptions). These rates are similar also to that reported (60%) in a 2003 study of PBS data.^12^

Greater proportions of TCA prescriptions by GPs may reflect off-label prescribing for other conditions such as insomnia, post-traumatic stress disorder, social phobia, panic disorder or chronic pain; they may also be due to familiarity with prescribing TCAs.

We found that approximately half of people in the analysis who were prescribed an antidepressant discontinued therapy for at least 6 months and did not return to treatment within the analysis period. In clinical practice, most clinicians would advise 9 to 12 months of pharmacotherapy for major depression; however, owing to a paucity of clinical trial data to inform the optimal length of treatment, there is no guideline for length of treatment. Despite this, persistence with antidepressant medication in this 10% PBS sample falls short of the advised time, with only a third of people persisting with antidepressant treatment for 12 months. This may reflect early discontinuation, or early treatment switch due to lack of response or tolerability concerns. Deprescribing may also occur, especially in older populations, owing to concerns about polypharmacy. Discontinuation may also be an attempt to regulate and clarify a prescribing plan for patients. Where adherence to antidepressant therapy is poor, a more structured plan is needed for follow-up to ensure treatment success.

Beyond 24 months, discontinuations plateaued, with a small proportion of people remaining on long-term antidepressants. However, antidepressant use in the longer term was lower than reported elsewhere. In a study from The Netherlands, chronic prescribing for 5 or more years occurs in up to four in ten people initially prescribed antidepressants.^13^

In recent years, there has been a growing concern regarding the over-prescription of antidepressants in Australia, which has the second highest level of prescribing in the Organisation for Economic Cooperation and Development overall.^14^ It remains unclear, however, whether this is simply because of the greater enthusiasm for pharmacotherapy of depression or whether it is partly because antidepressants are not stopped once started. The ready availability of a broad range of antidepressants means that the threshold for their prescription is now lower and patients have an opportunity to trial many more antidepressants than previously. Although this is described as possible overuse, the principal challenges remain the misuse of antidepressants and their potential to cause side-effects if used inappropriately or left unchecked.

### Limitations

As noted earlier in the Methods section, patients were excluded from this study if they were receiving mood stabilisers. These include lithium, the clinical use of which can be somewhat complicated as it is mainly and most widely used as a mood stabiliser.^15^ However, in practice it is also used for augmentation of the effect of an antidepressant. This means that a small proportion of those receiving lithium who were excluded may have had MDD.

Another limitation, often encountered in such analyses, is the fact that advice in the form of guidelines can change within the sampling frame. Indeed, during our window of 2013 to 2019, new guidelines were introduced. However, the advice was consistent, with the only key change being the introduction of new agents, and so we acknowledge this as a potential confounding factor.

This PBS 10% sample does not include medicines supplied to in-patients in public hospitals, those that are not listed on the PBS or those medications subsidised under the Repatriation PBS.^6^ The indication for prescription is not known for all medications included in the analysis, in particular, TCAs. The defined daily dose was also not available, and the assumption that an increase in dose strength corresponds to an increase in dose may have led to underestimation of the dose increase, as a patient may use a larger quantity of tablets from a lower strength formulation. Therefore, we recommend interpreting the results relating to dose change with caution. Furthermore, clinical information – for example, disease severity – was not available. It is also not known whether there were systematic differences in depressive presentation or in other clinical or demographic characteristics that may have affected the results of this study. Patients included in the study may have had previous episodes of MDD but had not received treatment with antidepressants in the 12 months prior to the study index episode. Finally, reasons for discontinuation, augmentation and continuation were not known. Therefore, it is unclear whether changes occurred owing to side-effects, lack of efficacy, achieving remission or other reasons. Although we excluded patients with prior use of antipsychotic agents, it is possible that augmentation of therapy occurred owing to a new-onset psychiatric illness.

PBS data may also be subject to seasonality due to the effect of stockpiling of medication towards the end of the year, when a family's spending on PBS listed medicines has reached the safety net threshold and the cost of subsequent PBS medicines are reduced to the concessional rate.

It is also important to note that the dosing and indication of antidepressants is not strictly adhered to; in addition to the obvious overlap between depression and anxiety (previously discussed), many antidepressants are used off-label to treat conditions such as insomnia and pain, especially in the case of TCAs. In addition, as the difficulty of treating depression increases and/or the longer the illness persists, there is an increasing tendency to use off-label medications to treat MDD and to resort to doses that are above those recommended. This is more likely to occur when treatment is administered by specialists as opposed to primary care physicians. Nevertheless, while acknowledging these limitations, our study provides some valuable insights as it allowed for long-term follow-up that is not feasible in clinical trials.

## Clinical implications

The treatment guidelines are likely to provide a useful reference framework, particularly for first-line treatment. The framework is less clearly followed where combination treatments and augmentation treatments are required, and in the most severe cases, where polypharmacy and off-label use are common in practice. It may not be possible to capture the many factors determining individual prescription patterns in a guideline, and this may to some extent explain why the prescribing patterns of GPs are different from those of psychiatrists. Given the complexity of the illness and its management, treatment should be tailored to the individual and guidelines should reflect the different presentations of depression in primary and specialist care settings.

## Data Availability

The data that support the findings of this study are available from Services Australia. Restrictions apply to the availability of these data, which were used under licence for this study.

## Supplementary material

For supplementary material accompanying this paper visit http://doi.org/10.1192/bjo.2022.522. click here to view supplementary material
