# Supplementary material for: Antidepressant prescribing patterns in Australia
Source: BJPsych Open. 2022 Jun 30;8(4):e120. doi: 10.1192/bjo.2022.522 (PMC9301763; doi:10.1192/bjo.2022.522)
Supplement: Supplementary file 1 [file S2056472422005221sup001.docx]

Supplementary Table 1 PBS indication restrictions for antidepressant medications

| Drug Name | ATC Code | PBS Item Code | Strength | Drug Class | Indication Restrictions |
| --- | --- | --- | --- | --- | --- |
| Amitriptyline | N06AA09 | 2417F | 10 mg | TCA | None listed |
| Amitriptyline | N06AA09 | 2418G | 25 mg | TCA | None listed |
| Amitriptyline | N06AA09 | 2429W | 50 mg | TCA | None listed |
| Citalopram | N06AB04 | 8702B | 10 mg | SSRI | Major depressive disorder |
| Citalopram | N06AB04 | 8703C | 40 mg | SSRI | Major depressive disorder |
| Citalopram | N06AB04 | 8220P | 20 mg | SSRI | Major depressive disorder |
| Clomipramine | N06AA04 | 1561E | 25 mg | TCA | Cataplexy &  obsessive-compulsive disorder & phobic disorders |
| Desvenlafaxine | N06AX23 | 10231L | 100 mg | SNRI | Major depressive disorder |
| Desvenlafaxine | N06AX23 | 10234P | 50 mg | SNRI | Major depressive disorder |
| Desvenlafaxine | N06AX23 | 10241B | 50 mg | SNRI | Major depressive disorder |
| Desvenlafaxine | N06AX23 | 10245F | 100 mg | SNRI | Major depressive disorder |
| Desvenlafaxine | N06AX23 | 9366Y | 50 mg | SNRI | Major depressive disorder |
| Desvenlafaxine | N06AX23 | 9367B | 100 mg | SNRI | Major depressive disorder |
| Dothiepin | N06AA16 | 1357K | 25 mg | TCA | None listed |
| Dothiepin | N06AA16 | 1358L | 75 mg | TCA | None listed |
| Doxepin | N06AA12 | 1011F | 10 mg | TCA | None listed |
| Doxepin | N06AA12 | 1012G | 50 mg | TCA | None listed |
| Doxepin | N06AA12 | 1013H | 25 mg | TCA | None listed |
| Duloxetine | N06AX21 | 9155W | 30 mg | SNRI | Major depressive disorder |
| Duloxetine | N06AX21 | 9156X | 60 mg | SNRI | Major depressive disorder |
| Escitalopram | N06AB10 | 10181W | 20 mg/mL | SSRI | Major depressive disorder & generalised anxiety disorder |
| Escitalopram | N06AB10 | 8700X | 10 mg | SSRI | Major depressive disorder |
| Escitalopram | N06AB10 | 8701Y | 20 mg | SSRI | Major depressive disorder |
| Escitalopram | N06AB10 | 9432K | 10 mg | SSRI | Generalised anxiety disorder & Social anxiety disorder |
| Escitalopram | N06AB10 | 9433L | 20 mg | SSRI | Generalised anxiety disorder & Social anxiety disorder |
| Fluoxetine | N06AB03 | 1434L | 20 mg | SSRI | Major depressive disorder & obsessive-compulsive disorder |
| Fluoxetine | N06AB03 | 8270G | 20 mg | SSRI | Major depressive disorder & obsessive-compulsive disorder |
| Fluvoxamine | N06AB08 | 8174F | 100 mg | SSRI | Major depressive disorder & obsessive-compulsive disorder |
| Fluvoxamine | N06AB08 | 8512B | 50 mg | SSRI | Major depressive disorder & obsessive-compulsive disorder |
| Imipramine | N06AA02 | 2420J | 10 mg | TCA | None listed |
| Imipramine | N06AA02 | 2421K | 25 mg | TCA | None listed |
| Mianserin | N06AX03 | 1627P | 10 mg | Tetracyclic | Severe depression |
| Mianserin | N06AX03 | 1628Q | 20 mg | Tetracyclic | Severe depression |
| Mirtazapine | N06AX11 | 8513C | 30 mg | NaSSA | Major depressive disorders |
| Mirtazapine | N06AX11 | 8855C | 15 mg | NaSSA | Major depressive disorders |
| Mirtazapine | N06AX11 | 8856D | 30 mg | NaSSA | Major depressive disorders |
| Mirtazapine | N06AX11 | 8857E | 45 mg | NaSSA | Major depressive disorders |
| Mirtazapine | N06AX11 | 8883M | 45 mg | NaSSA | Major depressive disorders |
| Mirtazapine | N06AX11 | 9365X | 15 mg | NaSSA | Major depressive disorders |
| Moclobemide | N06AG02 | 1900B | 150 mg | RIMA | Major depressive disorders |
| Moclobemide | N06AG02 | 8003F | 300 mg | RIMA | Major depressive disorders |
| Nortriptyline | N06AA10 | 2522R | 10 mg | TCA | Major depression - when other antidepressants have failed or contraindicated |
| Nortriptyline | N06AA10 | 2523T | 25 mg | TCA | Major depression - when other antidepressants have failed or contraindicated |
| Paroxetine | N06AB05 | 2242B | 20 mg | SSRI | Major depressive disorder & obsessive-compulsive disorder & panic disorder |
| Paroxetine | N06AB05 | 9197C | 20 mg | SSRI | Major depressive disorder & obsessive-compulsive disorder & panic disorder |
| Phenelzine | N06AF03 | 2856H | 15 mg | MAOI | Depression - when other antidepressants have failed or are inappropriate |
| Phenelzine | N06AF03 | 11713K | 15 mg | MAOI | Depression - when other antidepressants have failed or is inappropriate |
| Reboxetine | N06AX18 | 8583R | 4 mg | NRI | Major depressive disorders |
| Sertraline | N06AB06 | 2236Q | 50 mg | SSRI | Major depressive disorder |
| Sertraline | N06AB06 | 2237R | 100 mg | SSRI | Major depressive disorder |
| Sertraline | N06AB06 | 8836C | 50 mg | SSRI | Obsessive-compulsive disorder & panic disorder |
| Sertraline | N06AB06 | 8837D | 100 mg | SSRI | Obsessive-compulsive disorder & panic disorder |
| Tranylcypromine | N06AF04 | 2444P | 10 mg | MAOI | None listed |
| Venlafaxine | N06AX16 | 8301X | 75 mg | SNRI | Major depressive disorder |
| Venlafaxine | N06AX16 | 8302Y | 150 mg | SNRI | Major depressive disorder |
| Venlafaxine | N06AX16 | 8868R | 37.5 mg | SNRI | Major depressive disorder |
